# Impact of Nurse Manager’s Attributes on Multi-Cultural Nursing Teams: A Scoping Review

**DOI:** 10.3390/nursrep14030125

**Published:** 2024-07-15

**Authors:** Gisela Teixeira, Pedro Lucas, Filomena Gaspar

**Affiliations:** Nursing Research Innovation and Development Centre of Lisbon (CIDNUR), Nursing School of Lisbon, 1600-190 Lisbon, Portugal; prlucas@esel.pt (P.L.); mfgaspar@esel.pt (F.G.)

**Keywords:** cultural diversity, leadership, nurse manager, nursing team, review

## Abstract

Background: As global migration increases, nurse managers’ effectiveness in multi-cultural nursing work environments is crucial due to the rising cultural diversity within healthcare teams. Despite the increasing international recruitment of qualified nurses to address the worldwide nursing shortage, no studies have synthesised the impact of nurse managers’ attributes on nurses in multi-cultural nursing teams. Therefore, it was conducted a literature review aimed to synthesise the available literature on how nurse managers’ personality traits, competencies, behaviours, and leadership styles influence nurse outcomes in multi-cultural nursing teams. Methods: Scoping review conducted according to the Joanna Briggs Institute guidelines to map the relationship or influence of nurse managers’ personality traits, competencies, behaviours, and leadership styles on the outcomes of nurses in multi-cultural settings across various clinical environments. Searches were conducted across electronic databases such as CINAHL and MEDLINE, along with grey literature. Results: This review included 39 studies, highlighting 29 personality traits, 9 competencies, 115 behaviours, and 5 leadership styles that impact nurses’ outcomes. Key findings emphasise the importance of nurse managers being supportive, culturally competent, and effective communicators, with transformational leadership style being particularly beneficial. Conclusions: These findings provide insights for planning and developing training programmes to equip current and future nurse managers with skills to effectively lead in multi-cultural care settings.

## 1. Introduction

Healthcare systems worldwide face significant sustainability pressures, primarily due to the ageing population and, lately, the COVID-19 pandemic, which has uncovered the weaknesses of healthcare systems. The low retention of nurses and the widespread nursing shortage pose critical risks to patient safety [1], urgently requiring effective solutions to ensure safe and high-quality care in the face of future challenges that could further destabilise healthcare systems and threaten global health. The pandemic has exacerbated the shortage of nurses, prompting policy responses aimed at increasing the nurse supply at the system level worldwide [2]. In response, developed countries have strategically recruited qualified nurses internationally to address their human resource shortages [3,4]. This situation has led to the migration of qualified healthcare workers who are seeking not only better education, professional development, career progression, and improved wages, but also physical safety and health [5,6]. Consequently, the healthcare workforce is becoming increasingly multi-cultural.

Although the multi-cultural composition of teams might initially appear as a side-effect of healthcare worker migration, it presents an opportunity to deliver more person-centred care to the growing multi-cultural population. Rosa [7] emphasises the necessity for nursing human resources to be not only widely accessible and evenly distributed but also skilled and motivated to provide care that meets the socio-cultural needs and expectations of the diverse populations they serve. According to the World Health Organization [8], one of the global strategic goals for Human Resources in Health is, precisely, to invest in healthcare workers who can meet these needs through enhanced education and in supportive work environments [7], as well as by recruiting and retaining multi-cultural healthcare teams [9]. The practice of international recruitment has been notably prevalent in Gulf Cooperation Council countries [10], the United Kingdom, Ireland, the United States of America, Canada, and Australia [11].

The international recruitment of healthcare workers has increased interactions between managers and employees from various cultural backgrounds, sparking interest in trans-cultural leadership. Trans-cultural leadership is described as the act of developing, applying, and fostering a universal cultural vision, while cultivating multi-cultural synergy [12]. In nursing, this translates to a transformative process of culturally attuned behaviours, practices, and outcomes tailored to the cultural requirements of nurses and patients [13]. This leadership approach enables nursing teams to transcend cultural barriers, work collaboratively towards shared objectives, deliver culturally appropriate care, and enhance health outcomes for diverse populations [13]. Cunha [14] points out that trans-cultural leadership requires acumen in identifying and dealing with employees’ cultural characteristics, which can reinforce or mitigate the effects of a particular leadership style, and in adapting one’s behavioural profile to employees’ cultural expectations. The diversity of perspectives, experiences, and knowledge within culturally diverse work teams can foster innovative problem-solving behaviour but can also lead to conflicts, distrust, higher turnover, and lower job satisfaction and commitment [15], potentially resulting in a loss of innovation and organisational failure if not managed constructively [16]. 

Today’s nurse managers are challenged to promote culturally competent work environments [17], engaging nurses in organisational goals while addressing team needs [18], leading diverse teams effectively to enhance safe and culturally congruent care, and improving both patient and staff outcomes [13,19], as well as improving the financial performance of organisations that employ foreign-born workers [20]. Research shows that favourable nursing work environments can significantly enhance nurse satisfaction and retention, reduce burnout, and improve care quality and safety, ultimately leading to better patient experiences, reduced mortality, and lower organisational costs [21,22,23,24]. Therefore, employing qualified internationally recruited nurses will not address the nursing shortage problem, nor will it produce better outcomes for patients and organisations, unless there is a concerted effort to improve favourable nursing work environments. 

Although it is well documented that nurses’ leadership influences outcomes across patients, staff, and organisations, there remains a lack of comprehensive reviews on how nurse managers’ personality traits, competencies, behaviours, and leadership styles may impact the outcomes of a culturally diverse nursing workforce. This gap has led us to conduct this scoping review aimed at synthesising the best available evidence to date on this topic. Acknowledging this evidence is important for developing strategies to enhance the effectiveness of nurse managers in increasingly multi-cultural work environments.

## 2. Materials and Methods

This scoping review was conducted in accordance with the Joanna Briggs Institute guidelines for scoping reviews [25] and is reported following the recommendations of the Preferred Reporting Items for Systematic Reviews and Meta-Analyses Extension for Scoping Reviews (PRISMA-ScR) [26]. The initial research question for this study was “How does scientific evidence characterise the personality traits, behaviours, competencies, and leadership styles of nurse managers that influence outcomes in multi-cultural nursing teams?”. Accordingly, this review aimed to map the personality traits, competencies, behaviours, and leadership styles that influence the outcomes of multi-cultural nursing teams, based on scientific evidence.

### 2.1. Eligibility Criteria

▪ **Participants**: This scoping review included all papers focusing on nurses with differences in nationality, language, ethnicity, and/or religion within their nursing team, and on nurse managers who lead in multi-cultural settings. Papers were excluded if they did not identify cultural differences within the nursing team or if no nurse manager attributes were identified.
▪ **Concept**: This scoping review considered papers reporting personality traits, behaviours, competencies, and/or leadership styles of nurse leaders or managers and their association to at least one outcome affecting nurses in multi-cultural nursing teams. Outcomes included, but were not limited to, nurses’ professional satisfaction, motivation, teamwork, turnover, retention, absenteeism, professional performance, organisational commitment, conflicts, discrimination, violence, stress, burnout, well-being, cultural competence, and acculturation. Papers lacking identification of at least one impactful nurse manager attribute on any of these or other outcomes were excluded.
▪ **Context**: This scoping review included papers conducted in or reporting on several nursing care settings, including primary care, hospital care, long-term care, and social sectors.

### 2.2. Types of Sources

This scoping review included quantitative, qualitative, and mixed-methods study designs, literature reviews, master’s and doctoral theses and dissertations, text and opinion papers, books, and book chapters. 

### 2.3. Search Strategy

To locate relevant papers and documents for this review, both published and unpublished works were searched through electronic databases and grey literature. The electronic databases included:

- CINAHL (by EBSCO);
- MEDLINE (by EBSCO);
- Nursing & Allied Health Collection (by EBSCO);
- Cochrane Database of Systematic Reviews (by EBSCO);
- MedicLatina (by EBSCO);
- Psychology and Behavioral Sciences Collection (by EBSCO);
- SciELO;
- Wiley Online Library;
- LILACS;
- Scopus.

For grey literature, searches were conducted in the following databases:

- OpenGrey;
- RCAAP.

An initial exploratory search was conducted in the CINAHL and MEDLINE databases to identify synonymous search terms used in article indexing, titles, abstracts, and keywords. The terms used for this preliminary search were “nursing leadership”, “leadership traits”, “competencies”, “behaviours”, “skills”, “styles”, “multicultural nursing teams”, and “nurses’ outcomes”. The terms identified were then used to develop the full search strategy for each database (Supplementary File S1).

Full-text papers published in either Portuguese, English, or Spanish were included. Other languages were excluded due to the linguistic capabilities of the reviewers. No restrictions were placed on the publication date, as there had been no prior literature reviews on the subject, allowing access to the most comprehensive and relevant evidence available. 

The searches were conducted by two independent reviewers.

### 2.4. Paper Selection

**Figure 1:**
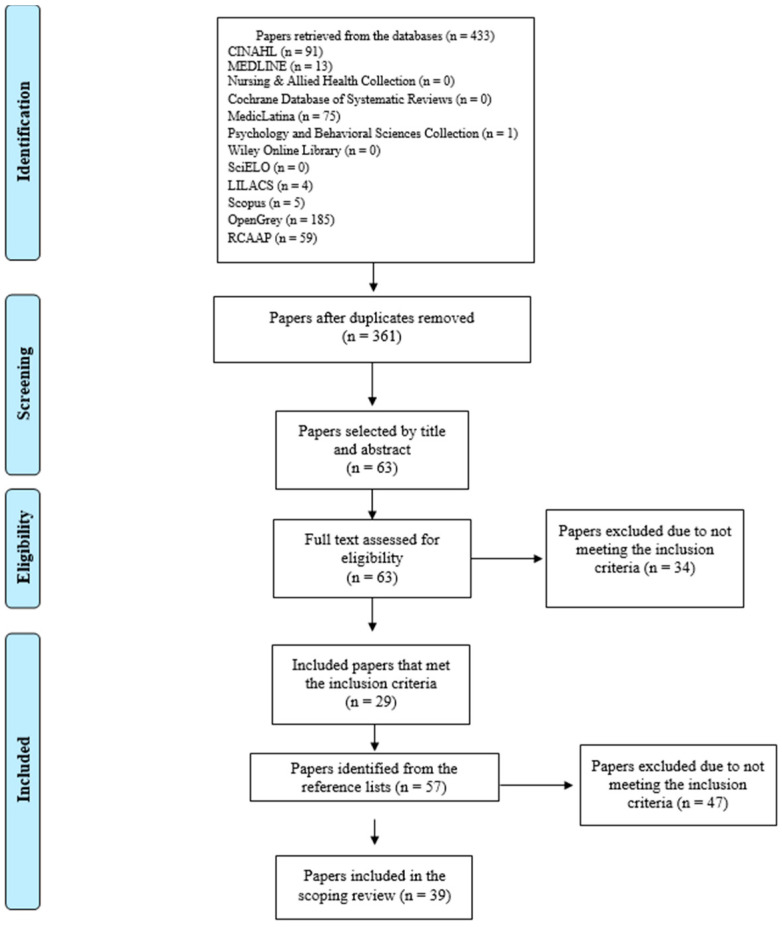
Flow diagram of the paper identification, selection, eligibility, and inclusion process.

The search strategy led to a total of 361 titles after the removal of duplicates. Their citations and abstracts were uploaded into Mendeley Desktop software (Version 1.19.8 © 2008–2020 Mendeley Ltd., London, UK) for bibliographic management and were organised according to the database from which they were retrieved. After screening the titles and abstracts, 64 papers were selected for full-text review. Of these, 29 met the inclusion criteria. The reference lists of these papers were then analysed to identify additional papers of interest based on the relevance of their titles. Fifty-seven papers were subsequently selected and analysed against the eligibility criteria. Finally, 39 papers were included in this scoping review. The process of paper identification, selection, eligibility, and inclusion is illustrated in the flow diagram below (Figure 1) as per Moher [27]. This process was conducted by two reviewers. Disagreements were resolved through consensus and, when necessary, consultation with a third reviewer.

### 2.5. Data Extraction

Data were extracted by two independent reviewers using a specifically developed data extraction tool. The information extracted included, whenever applicable, details such as the title, authors, year of publication, country, source of information, objectives, study design, participants, setting, research tools, and attributes of the nurse managers (personality traits, competencies, behaviours, and leadership styles). It also covered outcomes of nurses in multi-cultural nursing teams and the influence of nurse managers’ attributes on nurses’ outcomes. Since this scoping review did not aim to address overly specific research questions or assess the quality of the evidence produced, a critical appraisal of the methodological quality of the included papers was not performed [28].

## 3. Results

### 3.1. Characteristics of Included Papers

All papers were published between 1989 and 2023 in the USA (36%; n = 10), Saudi Arabia (25%; n = 9), the United Kingdom (11.1%; n = 4), Australia (8.3%; n = 3), the United Arab Emirates (8.3%; n = 3), Canada (5.6%; n = 2), or Finland (n = 2), and 1 each in Germany, Malaysia, Norway, Portugal, Singapore, and Israel in collaboration with the USA. Half (n = 20) were published in the last 10 years. They primarily reported quantitative and qualitative research, accounting for 36.1% (n = 13) and 25% (n = 12), respectively, along with text and opinion papers (25%; n = 9). Two papers reported mixed-method study designs, two reported systematic reviews of the literature, and one was published as an excerpt from a book chapter. Most participants in the research studies were nurse managers or leaders, and nurses of different nationalities or from different cultural backgrounds. In the reviewed literature, these nurses were identified in the papers as internationally educated, culturally and linguistically diverse, immigrant, foreign-born, expatriate, or overseas nurses. These terms are standardised as “culturally diverse nurses” throughout this scoping review.

### 3.2. Nurses’ Outcomes

Eighteen outcomes sensitive to the attributes of nurse managers were identified for nurses in multi-cultural teams. About 69% of the papers (n = 27) report nursing work-related outcomes, such as job satisfaction [29,30,31,32,33,34,35,36,37], organisational citizenship behaviours [35,38], organisational commitment [39,40,41,42], burnout [33], socio-cultural and professional integration [6,37,38,43,44,45,46,47,48,49], autonomy [38], professional development [45,50], retention [6,32,35,37,38,42,51,52,53,54], and continuous competence development [39]. Retention includes reports of both turnover and intention to leave. Socio-cultural and professional integration involves the process of transitioning and integrating culturally diverse nurses into new cultural and work environments. Professional development encompasses professional growth, career progression, and stagnation.

Approximately 38.5% of the papers (n = 15) describe outcomes related to the nursing team as a whole, such as teamwork [36,43], the achievement of common goals [38,55], performance [56,57,58], innovation and productivity [36], and interpersonal relationships [33,37,44,55,59,60,61,62,63]. The latter covers aspects of communication, conflicts, team motivation, cohesion, collaboration, mutual respect and understanding, empathy, and inclusion.

Following this, 23% of the papers (n = 9) report on psychosocial work climate, addressing issues like discrimination [6,37,38,45,50,64], coping with stress factors [64], fostering a culturally safe and competent work environment for nursing staff [43,65], and overall well-being [39].

Finally, five papers discuss care delivery-related outcomes, with three focusing on the delivery of culturally congruent care [37,63,66] and the remaining focusing on the quality of care provided [34,58].

### 3.3. Nurse Managers’ Attributes

The personality traits, competencies, behaviours, and leadership styles of nurse managers, which were found or expected to impact the outcomes of nurses in multi-cultural teams, were derived from the findings, perspectives, recommendations, and final considerations of the papers included in this review.

#### 3.3.1. Personality Traits

Twelve papers report personality traits, identifying twenty-eight desirable traits of nurse managers leading multi-cultural nursing teams. These include being supportive [34,38,46,53], cooperative [34,66], understanding [34], fair [34], proactive [34], unbiased [55], sensitive [38,54,59,60], approachable [38,53,66], respectful [53], an advocate for nurses [53], receptive to people and ideas [53], compassionate [38], empathetic [38], genuine [38], thoughtful [38], trustworthy [38,66], rigorous [38], insightful [60], focused [38], visionary [38], flexible [66], a good listener [66], courageous [66], self-confident [38,66], responsible [45,66], appreciative [64], aware of personal biases that affect leadership practices [63], and knowledgeable about personal cultural preferences that could influence leadership behaviour [63]. 

Supplementary File S2 synthesises all personality traits found in the literature and their impact on nurses’ outcomes in multi-cultural teams.

Traits such as being supportive, sensitive, and approachable are most frequently reported. A supportive, cooperative, and understanding nurse manager can improve nurses’ job satisfaction [34]. Supportive nurse managers are crucial in successfully transitioning culturally diverse nurses to new cultures and work environments [38,46], promoting a sense of belonging and thereby improving organisational citizenship behaviours and retention [38]. Dols et al. [53] noted that support from managers is the action most commonly requested by nurses of various ethnicities and generations in order to encourage their retention for an additional five years.

According to DeLellis [60], insight and sensitivity are vital for leaders in multi-cultural settings. Nurse managers who are sensitive to the unease of newcomers and recognise their eagerness to excel can foster team building and a cohesive workforce characterised by mutual respect and an understanding of each individual’s contributions to patient care [59]. This sensitivity also enhances nurse retention [54].

Approachability is another valued trait that nurses desire in their managers to facilitate their integration [38] and retention [53]. When facing challenges, nurses must feel that their manager is approachable [38]; otherwise, it can negatively affect communication and the quality of care by impacting their delivery of culturally congruent care [66].

One study highlights that a focused and visionary leader can significantly enhance the autonomy of culturally diverse nurses and the achievement of common goals [38].

Being biassed was reported as the only negative personality trait, and is of highest concern due to its adverse impact on nurses’ job satisfaction [34].

#### 3.3.2. Competences

Our review identified nine competencies essential for effectively managing multi-cultural nursing teams. These include cultural competence [32,36,38,43,44,54,55,60,61,63,65,66], effective communication [34,36,42,44,45,55,61,66], leadership skills [32,33,38,40,46,66], diversity management [49], problem and conflict solving [34,38,43,57,59], relational skills [34,38], coaching [38,46], supervision [56], and clinical expertise [38,53]. Supplementary File S3 synthesises the competencies of nurse managers identified in the literature and their impact on nurses’ outcomes in multi-cultural teams.

Cultural competence covers cultural awareness [55], knowledge of nurses’ cultural needs [32,43,54], the ability to recognise others’ cultural backgrounds as a source of knowledge [65], the ability to recognise the potential of nurses from ethnic minorities [65], the ability to recognise and connect cultural differences [61], the ability to understand the nature of culture and respect and how culture can manifest in an organisation [60], the ability to focus on cultural similarities [60], the ability to recognise that each person is not like any other person of the same cultural group [60], cultural sensibility [38], knowledge and understanding of the individual cultural influences among team members [63], and cultural intelligence [63]. Cultural sensitivity can motivate nurses to perform as best as they can and improve their organisational citizenship behaviours [38]. High levels of cultural intelligence may amplify the view of culture and diversity, for both leaders and staff, which builds innovative and productive teams and decreases bias-based decision making [63]. A visionary manager for the culturally congruent care contributes to its delivery to patients [66]. Cultural competence contributes to the retention of nurses from different cultural backgrounds [32,38,54] and enables host nurses to show empathy, contributing to a welcoming and culturally safe work environment that improves mutual understanding and relationships, teamwork, cohesion among groups of staff, and the achievement of common goals [43,55,63,65].

Effective communication implies speaking in clear and simple language [55], active listening [36,45], giving and asking for feedback and clarification [36], validating perceptions [36], understanding the subtle nuances in speech and body language [45], and being aware of other’s differing communication styles as well as one’s own [44]. The achievement of common goals is improved by managers who communicate clearly about tasks and what needs to be executed in order to perform them [55]. Nurses’ perceptions of leadership support depend on the type, frequency, and accuracy of manager’s communication, which can affect their satisfaction and retention [42]. Higher levels of manager’s communication and leadership skills lead to greater commitment and increased job satisfaction among nurses [34,42]. Effective communication eases nurses’ integration into the healthcare system, prevents discriminatory practices [45], and contributes to the delivery of culturally congruent care [66]. Both effective communication and cultural competence are critical for promoting better integration and teamwork among nurses [36,43].

According to Mitchell [33], managers must be qualified or have training in management and leadership skills in order to improve nursing staffing, enhance the safety of care, and mediate disputes, which affect nurses’ satisfaction and burnout in multi-cultural settings. Regarding Ncube’s findings [38], managers able to set goals and lead and motivate their team to achieve them can empower and strive nurses to achieve goals, and stimulate their autonomy and creativity. Organisational citizenship behaviours and retention might be improved in multi-cultural work environments when nurses identify their managers expressing individualised consideration [38]. Leaders that demonstrate higher scores of inspirational motivation seem to lead more committed nurses as well [40]. Goh and Lopez [32] observed that the use of nursing informatics in clinical fields can lead to lower levels of job satisfaction among nurses due to the effort of learning the new technology while trying to adapt to a new work environment. A supportive leadership is essential for this transition and integration into the healthcare work environment [46]. Nursing work environments are a predictor of migrant nurses’ intentions to leave; thus, nurse managers must have the ability to lead a ward and the practice environment to improve retention [32], which includes supporting nurses´ learning curves regarding information systems. Amouri and O’Neill [66] emphasise the need for knowledge of leadership styles, the ability to motivate and appreciate nurses, and the need for a transformational leadership style to ensure the delivery of culturally competent care.

According to Kamau et al. [49], incorporating diversity leadership skills in educational programmes for nurse leaders and managers can enhance their understanding and management of workforce diversity, promoting the successful employment and integration of culturally and linguistically diverse nurses.

Managers must effectively handle conflicts arising from cultural clashes and racism within multi-cultural work environments [38,43]. When cultural clashes occur, the manager is expected to act as a facilitator to arrive at a reasonable resolution for both parties [59], contributing to both positive interpersonal relationships and culturally safer work environments [43]. According to Ancarani et al. [57], an inverse relationship exists between religious diversity and ward efficiency. This relationship is moderated by several factors, including the ability to manage task conflicts.

Relational skills include transformational skills and the capacity to foster good relationships with all staff members. Nurses who perceive their managers as relationship-oriented tend to report higher job satisfaction [34]. Furthermore, one study indicates that transformational skills, among other factors, facilitate a successful transition into the healthcare environment [38].

Effective communication, conflict resolution, and the ability to cultivate good relationships are key competencies. All managers should receive training in these areas to build trust within the team, which is crucial for enhancing job satisfaction [34].

Coaching and supervision have been identified as key competencies necessary for leading a multi-cultural nursing team. Coaching is essential for adequately supporting the needs of culturally diverse nurses and facilitating their integration into clinical settings [38,46]. This includes recognising the individual learning times of nurses [46]. Additionally, supervisory skills are vital for providing support, empowering staff, and motivating them to enhance their performance [56].

From the perspective of nurses from different generations and ethnicities, it is essential for nurse managers to possess clinical expertise, including reasoning abilities and clinical competences [53], which contribute to enhancing nurses’ integration into the work environment [38].

#### 3.3.3. Behaviours

Thirty-two papers examined the expected or observed behaviours (attitudes, decisions, and practices) of nurse managers when leading multi-cultural teams. They documented 115 behaviours, of which 83.5% (n = 96) were beneficial and 16.5% (n = 19) were unfavourable for nurses’ outcomes. These 115 behaviours of nurse managers leading multi-cultural nursing teams were used in the development of the Portuguese Transcultural Nursing Leadership Questionnaire [67].

Beneficial behaviours were organised into nine categories: (a) appreciating and recognising, (b) improving practice, (c) sharing governance, (d) advancing nurses’ careers, (e) integrating into the work environment, (f) promoting psychosocial well-being at work, (g) dynamising groups, (h) communicating, and (i) coordinating nursing care. Nurse managers’ behaviours identified as negative for nurses in multi-cultural nursing teams were organised into five categories: (a) downgrading, (b) lacking management performance, (c) centralising governance, (d) favouring, and (e) hampering nurses’ career advancement. Supplementary File S4 synthesises the impact of these categories of behaviours on nurses’ outcomes in multi-cultural teams. The following paragraphs describe the contents of these categories with examples of the detailed behaviours.

Promoting psychosocial well-being at work had the highest number of beneficial behaviours. It included developing and implementing policies and initiatives that recognise, respect, value, support, and leverage the benefits of diversity; investigating incidents related to physical or verbal abuse and discriminatory or harassing behaviours; and implementing measures to decrease the risk of reoccurrence. Among others, these behaviours improve socio-cultural and professional integration [6,43,46,48], interpersonal relationships [60,61,63], teamwork [43], and retention [6]; fight discrimination [45]; increase satisfaction [33]; foster a culturally safe and competent work environment [65]; and benefit the delivery of bias-free care [63].

Appreciating and recognising nurses for their work may be one of the most significant gestures that contributes to job satisfaction [33]. Practices such as making regular rounds to the units to thank the staff, recognising their knowledge and professional skills and maximising these in clinical practice, providing fringe benefits and contingent rewards, and allocating nurses to their clinical areas of expertise, among other strategies, improve job satisfaction [31,32,33]. These actions also facilitate socio-cultural and professional integration [6,38,46], enhance interpersonal relationships [63], help nurses to cope with stress factors [64], may improve performance [56], increase retention [32,38,52,53], and enhance the delivery of culturally congruent care [66].

Behaviours that improve nursing practice—such as building educational and training programmes, evaluating and monitoring the outcomes of professional learning in scope and effectiveness, and providing supervision and feedback—promote socio-cultural and professional integration [6,46], improve interpersonal relationships [44], improve professional development [33], may increase job satisfaction [30], enhance performance [58], and improve culturally congruent care delivery [66].

Sharing governance strategies, like involving nurses in hospital affairs and decision making and delegating nurses to participate in nursing and hospital committees or teams within the workday schedule, enhance job satisfaction [31,33,34]. Supporting, appreciating, and involving nurses in decisions makes them more committed and satisfied, and, consequently, leads to higher levels of performance [56].

Behaviours aimed at advancing nurses’ careers, such as reviewing equality, diversity, recruitment, or career development policies and encouraging individuals from various ethnic and cultural groups to develop their leadership skills, are recognised not only for improving professional development [33,45] but also for enhancing job satisfaction [31]. Since these behaviours combat discriminatory practices [45], they also foster interpersonal relationships [33], contribute to a culturally safe and competent work environment, and enhance the delivery of culturally congruent care [65,66].

Supporting nurses’ integration into the work environment through strategies like preceptorship or regular meetings with newly hired culturally diverse nurses can facilitate socio-cultural and professional integration [6,38,43,45,46], improve interpersonal relationships [59,62], enhance retention [6], and boost teamwork [43].

Strategies for group dynamics, such as organising regular group meals to socialise and promote team spirit or creating spaces and opportunities for nurses to voluntary share their personal experiences, are described as facilitators of interpersonal relationships [44,59], socio-cultural and professional integration [48], and teamwork [43]. These activities also improve job satisfaction [31] and culturally congruent care practices [66].

Communicating directly with nurses, validating different perspectives, encouraging questioning, and using clear and simple language are just a few examples of communication-related behaviours that improve job satisfaction [33], facilitate socio-cultural and professional integration [38,44,46], enhance interpersonal relationships [44,59], aid in coping with stress factors [64], and contribute to the achievement of common goals [55].

Behaviours related to nursing care coordination—such as, among other strategies, establishing clear policies for patient assignments, schedules, evaluations, annual leave, and promotions; decentralising tasks; implementing a working method based on the knowledge of patients and nurses’ biases; and explaining to culturally diverse nurses the role of the family in patient care in the dominant culture—improve nurses’ satisfaction [34], enhance interpersonal relationships among staff [62], decrease burnout [33], improve the quality of care [58], and promote bias-free and culturally congruent care [63,66].

Downgrading behaviours, such as undervaluing nurses and not recognising their achievements or professional skills, among other actions listed, lead to dissatisfaction [30,51], decrease teamwork and productivity [36], increase the intention to leave [51], and foster feelings of discrimination [6,45].

Lacking performance management, characterised by not providing adequate supervision and feedback on the performance of culturally diverse nurses, primarily reduces satisfaction [30].

Not involving nurses in decision making and discouraging their improvement are behaviours that centralise units’ or hospitals’ governance, which are unfavourable for nurses’ satisfaction [30] and retention [35].

Managers displaying favouritism—such as by spending more time with nurses from cultures that they most identify with, planning schedules and vacations unequally among nurses of different cultural backgrounds, or making unequal decisions related to the method for nursing care delivery—increases dissatisfaction [34], discrimination [6,38,64], and turnover [52]; harms teamwork and productivity [36]; and harms professional development [50].

Promoting nurses based on cultural criteria rather than merit or lacking transparency in promotion processes are unfavourable practices that impact retention [52], hinder professional development [50], and perpetuate discrimination within organisations [6].

Experiences of discrimination from managers, such as de-skilling competencies, assigning the same nurses to the most care-dependent patients, targeting them with undesirable shifts, planning off-days and vacations unfairly, and promoting colleagues based on cultural criteria rather than merit [38,45,64], are highlighted as factors that complicate the transition to workplaces in host countries [6].

#### 3.3.4. Leadership Styles

Five different leadership styles were identified within multi-cultural nursing work environments—preferential [34], relational [34], autocratic [52], transformational [29,35,38,40,41,66], transactional [29,38,41,66], and formal [49]. Table 1 synthesises the impact of nurse managers’ leadership styles on nurses’ outcomes.

Preferential and relational leadership styles are observed when nurse managers, respectively, treat nurses from the same country of origin more favourably, and when there is cooperation between ward nurses and the nursing leadership team to produce the best possible patient outcomes [34]. The preferential leadership style conveys a degree of favouritism and promotes dissatisfaction and feelings of unfairness among team members, which can lead to reduced engagement, poor staff retention, and poor motivation, consequently affecting the quality of patient care [34]. Conversely, the relational leadership style is highlighted as fostering nurses’ satisfaction and improving the quality of care they provide [34].

Both the transformational and transactional leadership styles have been reported as positive for nurses’ job satisfaction [29,35] and organisational commitment [41], with transformational showing a stronger correlation. According to one study [66], the characteristics of these two leadership styles are essential for developing the ability of nursing staff to seek opportunities that enhance their skills in providing effective and culturally congruent care. Nurses in multi-cultural teams exhibit higher levels of organisational citizenship behaviours, recognise the effectiveness of their nurse managers, and feel more satisfied when they perceive their leadership as transformational [35]. Nurses feel safe and develop a sense of belonging when they observe their leader exhibiting people-centred and values-based leadership behaviours, such as transformational leadership [38].

The autocratic leadership style negatively influences culturally diverse nurses’ decision to leave [52]. In contrast, transformational nurse leaders are more likely to be successful in building a positive work environment, thereby increasing retention [35].

Formal leadership enables nurse leaders to adopt effective leadership styles, act with competence, and successfully integrate culturally diverse nurses [49].

## 4. Discussion

The quality of leaders’ interpersonal skills, their role in promoting favourable working conditions, their knowledge of patients’ needs, and their engagement in behaviours that inspire nursing teams to higher performance levels are significant predictors of improved patient outcomes [68,69]. Several studies have examined the personality traits, behaviours, competencies, and leadership styles of nurse managers that influence both nurses and patients’ outcomes. Traits such as extraversion; agreeableness; conscientiousness; neuroticism; openness to experience, temperament, needs, motives, and values; integrity; a sense of moral purpose; and emotional maturity were identified as influential to the managerial competence of first-line managers [70]. The evidence suggests that relational leadership styles, including transformational leadership, are positively associated with patient outcomes such as increased patient satisfaction, lower mortality rates, and reduced medication errors and hospital-acquired infections [69]. Additionally, transformational leadership is preferred for promoting positive work environments and nursing workforce outcomes, such as job satisfaction, retention, and individual productivity [71]. Effective nurse managers are crucial for maintaining a supportive work environment and ensuring high-quality patient care, as they possess essential competencies such as emotional intelligence, communication, and leadership skills [68]. Despite this evidence of personality traits, competences, and leadership styles, none of these studies specifically address the multi-cultural dimension within nursing teams. While most of the characteristics of effective nursing management remain consistent, the context of cultural diversity introduces unique challenges. For instance, cultural competence and sensitivity to diverse cultural backgrounds are critical in preventing misunderstandings and fostering a cohesive work environment in multi-cultural teams, as identified in our review. Additionally, studies on nursing in general do not highlight culturally congruent care as an outcome of nursing management and leadership.

Acknowledging the weight of multi-cultural aspects within nursing teams is essential in order to address the specific needs and challenges that arise in diverse work environments. Multi-cultural teams often face communication barriers, cultural misunderstandings, and varied expectations based on cultural backgrounds. Therefore, nurse managers must not only exhibit general leadership qualities but also possess cultural competence and the ability to navigate and mediate cultural differences effectively. Therefore, this scoping review provides a comprehensive overview of the attributes of nurse managers leading specifically multi-cultural nursing teams, and their impact not only on nurses’ outcomes but also indirectly on patients, particularly in the quality and delivery of culturally congruent care.

The analysed evidence reports the necessity of having supportive, culturally competent, and transformational leaders who are effective communicators in order to maximise outcomes in multi-cultural nursing work environments. Behaviours that appreciate and recognise, and contribute, to psychosocial well-being at work also significantly affect the most nurses’ outcomes. 

Managers have a responsibility to support and serve, requiring competence and knowledge about how to assist nurses when challenging situations in clinical nursing cause distress [72]. Transitioning to a multi-cultural nursing work environment presents challenges that can lead to suffering among nurses due to cultural and linguistic differences and discriminatory practices, resulting in dissatisfaction and low retention within these environments. These serious issues, which strain the efficiency of healthcare systems, can be moderated by managers who support, recognise, and value nurses’ performance, listen to their team’s problems, and negotiate solutions [52]. Organisational efforts to strengthen nurses’ autonomous motivation through supportive supervisors and coworkers’ behaviours are necessary for contributing to a well-established workforce in the nursing profession [73], and for improving satisfaction and retention.

The increasing diversity of human resources demands management based on teams’ characteristics, prioritising cultural competence as a mandatory skill for contemporary managers [74]. This is vital for diversity management [75] and promoting healthy work environments within healthcare organisations [76,77]. Cultural competence is considered an organisational attribute that facilitates the professional practice of nursing teams in multi-cultural work environments and contributes to improving patient care. Therefore, its development should be considered at all organisational levels [17]. It enhances individuals’ self-awareness and their awareness of others, allows individuals to understand different worldviews, and helps in identifying differences and similarities relevant to planning culturally congruent care and management practice [78]. Dauvrin and Lorant [79] observed that leaders’ levels of cultural competence partially contribute to healthcare workers’ cultural competence, which, in turn, positively affects their satisfaction and retention [80], the quality of care they provide, and patients’ health, safety, and satisfaction [81,82], reinforcing our findings. As Teixeira et al. [17] stated, it is crucial for nurse managers to engage in the process of becoming culturally competent in order to better understand the needs of the patients they serve and to effectively lead culturally diverse nursing teams.

Suarez-Balcazar et al. [83] also highlight the organisation’s role in supporting practitioners’ efforts to engage in culturally appropriate practices, which includes assessing whether cultural competence is reflected in the workplace’s mission statement, policies, and procedures. Another study [84] suggests that human resource managers in culturally diverse organisations should prioritise openness to diversity through diversity training and reward group-oriented behaviours. These strategies support our listed behaviours of developing policies and initiatives that recognise, respect, value, support, and leverage the benefits of diversity, and implementing a reward system that encourages demonstrations of interpersonal and cross-cultural respect.

Effective communication from nurse managers is fully supported by RNAO [76]. Communication within healthcare organisations is directly or indirectly related to patient care, and, as stated by Mosed et al. [85] any “miscommunication due to a lack of understanding of culture can result in confusion leading to negative health outcomes” (p. 997). Barriers such as different languages and their characteristics [64] can negatively affect patients’ perceived quality of care, safety, and satisfaction [86], may limit nurses’ understanding of the healthcare system they work for, and may make patient-centred care unfeasible [87]. Therefore, nurse managers’ communication must be effective to ensure patient safety. In work environments where verbal and non-verbal communication may vary significantly, it is important to listen actively, promote an open-door policy, meet face-to-face, and never assume that a message is understood without validation [88]. These are strategies compatible with our listed behaviours of improving communication.

Transformational leadership was reported as the style that improves the most outcomes, namely job satisfaction, retention, organisational citizenship behaviours, and commitment, confirming the prevailing literature [71]. According to Ashikali et al. [89] and McCutcheon [90], transformational leadership appears to best mitigate the negative consequences of teams’ diversity and enhance staff satisfaction, cooperation, teamwork, inclusion, cohesion, and group performance. No other studies were found to support the relevance of transformational leaders in improving culturally congruent care. Therefore, given the increasing diversity among patients and healthcare workers, the moderating effects of nurse managers’ leadership practices on nursing care delivery and their impacts on patients’ outcomes in multi-cultural nursing work environments must be explored in future research.

A biassed nurse manager who displays downgrading and favouring behaviours and adopts a preferential-based leadership style worsens a significant number of outcomes for their nurses. Favouritism practices related to relationships, unit decisions, and promotions are common problems within multi-cultural nursing work environments, leading to dissatisfaction and an intention to leave. This is supported by a study that found that higher levels of favouritism were associated with an increased nurse turnover and nurses’ intention to leave [91]. However, our results related to discrimination and favouring behaviours are primarily derived from bedside nurses’ perceptions, who may also be biassed regarding their managers’ profiles in multi-cultural settings. Yukl [15] states that “just as leaders tend to be biased toward making internal attributions about followers, followers seem to have a bias toward making internal attributions about leaders” (p. 232).

While general best practices in nursing management are crucial, addressing the nuances of multi-cultural teams ensures a more inclusive and effective approach to nursing leadership.

## 5. Limitations and Recommendations

This review provides insights into the personality traits, competencies, behaviours, and leaderships styles of nurse managers that may be developed or avoided to improve the outcomes of nurses in multi-cultural teams. We believe that the exhaustive search strategy defined for each database and the eligibility criteria were of high relevance to differentiate our findings. However, in multi-cultural work environments, no single leadership style is considered equally effective for employees of different cultural backgrounds, since each individual has unique expectations and perceptions of leadership effectiveness [92]. Therefore, a nurse’s cultural background can shape their expectations of effective leadership behaviours and the necessary traits and competencies of nurse managers, complicating their identification of an idealised nurse manager profile in multi-cultural nursing work environments. We recommend conducting future comparative research to examine differences between multi-cultural and non-multi-cultural nursing teams in order to identify unique challenges and develop tailored best practices.

## 6. Conclusions

The nursing shortage, coupled with the growing demands of an ageing population, is an escalating reality in developed countries. Recruiting culturally diverse nurses not only addresses this challenge but also presents an opportunity to enhance creativity and innovation, as well as to improve the quality of culturally congruent care for patients from different cultural backgrounds. Managers must be attuned to the cultural differences and unique needs of their staff, understanding how their own personality traits, competencies, behaviours, and leadership styles impact their nurses. This understanding is crucial to attract and retain skilled, productive, and satisfied nurses, ultimately leading to improved patient outcomes.

Our findings can inform the planning and development of training programmes that equip current and future nurse managers with skills to effectively lead in multi-cultural care settings. Additionally, these insights may provide guidelines for recruiting and selecting candidates for nursing management positions that are capable of effectively managing diversity in order to promote healthy work environments.

## Figures and Tables

**Table 1:** Impact of nurse managers’ leadership styles on nurses’ outcomes.

Nurse Managers’ Leadership Styles	Nurses’ Outcomes						
	Job Satisfaction	OCB	Organisational Commitment	Retention	Socio-Cultural and Professional Integration	CCC	Quality of Care
Preferential	−						
Transactional	+		+			+	
Transformational	+	+	+	+		+	
Relational	+						+
Autocratic				−			
Formal					+		

Note: ‘+’ means positive impact; ‘−’ means negative impact. Abbreviations: CCC—culturally congruent care; OCB—organisational citizenship behaviours.

## Supplementary Materials

The following supporting information can be downloaded at https://www.mdpi.com/2039-4403/14/3/125/s1, Supplementary File S1: Full search strategy; Supplementary File S2: Nurse managers’ personality traits impact on nurses’ outcomes in multi-cultural teams; Supplementary File S3: Impact of nurse managers’ competences on nurses’ outcomes in multi-cultural teams; Supplementary File S4: Impact of nurse managers’ behaviours on nurses’ outcomes in multi-cultural teams.

## Public Involvement Statement

There was no public involvement in any aspect of this research.

## Guidelines and Standards Statement

This manuscript was drafted against the Preferred Reporting Items for Systematic Reviews and Meta-Analyses Extension for Scoping Reviews (PRISMA-ScR) [26].

## Abbreviations

AONL: American Organization for Nursing Leadership; RNAO: Registered Nurses’ Association of Ontario; USA: United States of America.

## References

[1] Lake E. (2007). The nursing practice environment: Measurement and evidence. Med. Care Res. Rev..

[2] Buchan J., Catton H., Shaffer F. (2022). Sustain and Retain in 2022 and Beyond.

[3] Ohr S.O., Holm D., Brazil S. (2016). The transition of overseas qualified nurses and midwives into the Australian healthcare workforce. Aust. J. Adv. Nurs..

[4] Kelly S., Fowler C. (2019). Enhancing the recruitment and retention of overseas nurses from Kerala, India. Nurs. Stand..

[5] Kingma M. (2001). Nursing migration: Global treasure hunt or disaster-in-the-making ?. Nurs. Inq..

[6] Newton S., Pillay J., Higginbottom G. (2012). The migration and transitioning experiences of internationally educated nurses: A global perspective. J. Nurs. Manag..

[7] Rosa W. (2017). Global health and global nursing: Emerging definitions and directions. A New Era in Global Health: Nursing and the United Nations 2030 Agenda for Sustainable Development.

[8] World Health Organization (2020). State of the World’s Nursing 2020: Investing in Education, Jobs and Leadership.

[9] Breaky S., Evans L., Rosa W. (2017). Global health ethics. A New Era in Global Health: Nursing and the United Nations 2030 Agenda for Sustainable Development.

[10] Khoja T., Rawaf S., Qidwai W., Rawaf D., Nanji K., Hamad A. (2017). Health Care in Gulf Cooperation Council Countries: A Review of Challenges and Opportunities. Cureus.

[11] Rosenkoetter M.M., Nardi D., Bowcutt M. (2017). Internationally Educated Nurses in Transition in the United States: Challenges and Mediators. J. Contin. Educ. Nurs..

[12] Foulkes R. (1994). Transcultural leadership: Empowering the diverse workforce. Columbia J. World Bus..

[13] Teixeira G., Cruchinho P., Lucas P., Gaspar F. (2023). Transcultural nursing leadership: A concept analysis. Int. J. Nurs. Stud. Adv..

[14] Cunha M., Rego A., Cunha R., Cabral-Cardoso C., Editora R.H. (2007). Manual de Comportamento Organizacional e Gestão.

[15] Yukl G. (2013). Leadership in Organizations.

[16] Parvis L. (2003). Diversity and Effective Leadership in Multicultural Workplaces. J. Environ. Health.

[17] Teixeira G., Gaspar F., Lucas P. (2022). Nurse manager’s role in promoting culturally competent work environments in nursing: An integrative review. New Trends Qual. Res..

[18] Nunes E.M.G.T., Gaspar M.F.M. (2017). Quality of the leader-member relationship and the organizational commitment of nurses. Rev. Esc. Enferm. USP.

[19] Russell G.P. (2022). President’s Message: National Nurses Month-The Importance of Recognizing Transcultural Nursing Leaders. J. Transcult. Nurs..

[20] Thekdi P., Wilson B., Xu Y. (2011). Understanding post-hire transitional challenges of foreign-educated nurses. Nurs. Manag..

[21] Al-Ghraiybah T., Sim J., Lago L. (2021). The relationship between the nursing practice environment and five nursing-sensitive patient outcomes in acute care hospitals: A systematic review. Nurs. Open.

[22] Lucas P., Jesus E., Almeida S., Araújo B. (2021). Validation of the Psychometric Properties of the Practice Environment Scale of Nursing Work Index in Primary Health Care in Portugal. Int. J. Environ. Res. Public Health.

[23] Lake E., Sanders J., Duan R., Riman K., Schoenauer K., Chen Y. (2019). A Meta-Analysis of the Associations between the Nurse Work Environment in Hospitals and 4 Sets of Outcomes. Med. Care.

[24] Ambani Z., Kutney-Lee A., Lake E. (2020). The nursing practice environment and nurse job outcomes: A path analysis of survey data. J. Clin. Nurs..

[25] Peters M., Godfrey C., McInerney P., Munn Z., Tricco A., Khalil H., Aromataris E., Munn Z. (2020). Scoping Reviews (2020 version). JBI Manual for Evidence Synthesis.

[26] Tricco A.C., Lillie E., Zarin W., O’Brien K.K., Colquhoun H., Levac D., Moher D., Peters M.D.J., Horsley T., Weeks L. (2018). PRISMA Extension for Scoping Reviews (PRISMA-ScR): Checklist and Explanation. Ann. Intern. Med..

[27] Moher D., Liberati A., Tetzlaff J., Altman D.G. (2009). Preferred Reporting Items for Systematic Reviews and Meta-Analyses: The PRISMA Statement. PLoS Med..

[28] Arksey H., O’Malley L. (2005). Scoping Studies: Towards a Methodological Framework. Int. J. Soc. Res. Methodol..

[29] Ahmad A., Adi M., Noor H., Rahman A., Yushuang T. (2013). The influence of leadership style on job satisfaction among nurses. Asian Soc. Sci..

[30] Almalki M.J., Fitzgerald G., Clark M. (2012). Quality of work life among primary health care nurses in the Jazan region, Saudi Arabia: A cross-sectional study. Hum. Resour. Health.

[31] Itzhaki M., Ea E., Ehrenfeld M., Fitzpatrick J.J. (2013). Job satisfaction among immigrant nurses in Israel and the United States of America. Int. Nurs. Rev..

[32] Goh Y.S., Lopez V. (2016). Job satisfaction, work environment and intention to leave among migrant nurses working in a publicly funded tertiary hospital. J. Nurs. Manag..

[33] Mitchell J. (2009). Job Satisfaction and Burnout among Foreign-Trained Nurses in Saudi Arabia: A Mixed-Method Study.

[34] Saleh U., O’Connor T., Al-Subhi H., Alkattan R., Al-Harbi S., Patton D. (2018). The impact of nurse managers’ leadership styles on ward staff. Br. J. Nurs..

[35] Suliman W.A. (2009). Leadership styles of nurse managers in a multinational environment. Nurs. Adm. Q..

[36] Taylor R. (1998). Check your cultural competence. Nurs. Manag..

[37] Teixeira G., Lucas P., Gaspar F. (2022). International Portuguese Nurse Leaders’ Insights for Multicultural Nursing. Int. J. Environ. Res. Public Health.

[38] Ncube E. (2017). Influence of Leadership Styles on Expatriate Nurses’ Professional Integration in the UAE. Ph.D. Thesis.

[39] Kiviniitti N., Kamau S., Mikkonen K., Hammaren M., Koskenranta M., Kuivila H., Kanste O. (2023). Nurse leaders’ perceptions of competence-based management of culturally and linguistically diverse nurses: A descriptive qualitative study. Nurs. Open.

[40] Al-Yami M., Galdas P., Watson R. (2018). Leadership style and organisational commitment among nursing staff in Saudi Arabia. J. Nurs. Manag..

[41] Dahshan M.E., Youssef H.A.M., Aljouaid M., Babkeir R.A., Hassan W.B. (2017). Effect of Nurse Managers’ Leadership Styles on Organizational Commitment of Nurses Working at Taif Governmental Hospitals in Kingdom of Saudi Arabia. Glob. J. Manag. Bus. Res..

[42] Fowler K. (2018). Communicating in a culturally diverse workforce. Nurs. Manag..

[43] Chen L., Xiao L., Han W., Meyer C., Müller A. (2020). Challenges and opportunities for the multicultural aged care workforce: A systematic review and meta-synthesis. J. Nurs. Manag..

[44] Gill G.K., McNally M.J., Berman V. (2018). Effective diversity, equity, and inclusion practices. Healthc. Manag. Forum.

[45] Hunt B. (2007). Managing equality and cultural diversity in the health workforce. J. Clin. Nurs..

[46] Sherman R., Eggenberger T. (2008). Transitioning internationally recruited nurses into clinical settings. J. Contin. Educ. Nurs..

[47] Takeno Y. (2010). Facilitating the transition of Asian nurses to work in Australia. J. Nurs. Manag..

[48] Xiao L.D., Willis E., Jeffers L. (2014). Factors affecting the integration of immigrant nurses into the nursing workforce: A double hermeneutic study. Int. J. Nurs. Stud..

[49] Kamau S., Oikarainen A., Kiviniitty N., Koskenranta M., Kuivila H., Tomietto M., Kanste O., Mikkonen K. (2023). Nurse leaders’ experiences of how culturally and linguistically diverse registered nurses integrate into healthcare settings: An interview study. Int. J. Nurs. Stud..

[50] Henry L. (2007). Institutionalized disadvantage: Older Ghanaian nurses’ and midwives’ reflections on career progression and stagnation in the NHS. J. Clin. Nurs..

[51] Al-Ahmadi H. (2014). Anticipated nurses’ turnover in public hospitals in Saudi Arabia. Int. J. Hum. Resour. Manag..

[52] Alshareef A.G., Wraith D., Dingle K., Mays J. (2020). Identifying the factors influencing Saudi Arabian nurses’ turnover. J. Nurs. Manag..

[53] Dols J.D., Chargualaf K.A., Martinez K.S. (2019). Cultural and generational considerations in RN retention. J. Nurs. Adm..

[54] Gullatte M.M., Jirasakhiran E.Q. (2005). Retention and recruitment: Reversing the order. Clin. J. Oncol. Nurs..

[55] Alexis O. (2005). Managing change: Cultural diversity in the NHS workforce. Nurs. Manag..

[56] Al-Ahmadi H. (2009). Factors affecting performance of hospital nurses in Riyadh Region, Saudi Arabia. Int. J. Health Care Qual. Assur..

[57] Ancarani A., Ayach A., Di Mauro C., Gitto S., Mancuso P. (2016). Does religious diversity in health team composition affect efficiency? Evidence from Dubai. Br. J. Manag..

[58] Thiederman S. (1989). Managing the foreign-born nurse. Nurs. Manag..

[59] Burner O.Y., Cunningham P., Hattar H.S. (1990). Managing a multicultural nurse staff in a multicultural environment. J. Nurs. Adm..

[60] DeLellis A.J. (2006). Leadership for cross-cultural respect among health care personnel: An alternative approach. Health Care Manag..

[61] Mortell S. (2013). Delving into diversity-related conflict. Nurs. Manag..

[62] Munkejord M.C. (2019). Challenging the ethnic pyramid: Golden rules and organisational measures towards a more inclusive work environment. J. Nurs. Manag..

[63] Richard-Eaglin A. (2021). The Significance of Cultural Intelligence in Nurse Leadership. Nurse Lead..

[64] Schilgen B., Handtke O., Nienhaus A., Mösko M. (2019). Work-related barriers and resources of migrant and autochthonous homecare nurses in Germany: A qualitative comparative study. Appl. Nurs. Res..

[65] Chambers C., Alexis O. (2004). Creating an inclusive environment for black and minority ethnic nurses. Br. J. Nurs..

[66] El Amouri S., O’Neill S. (2014). Leadership style and culturally competent care: Nurse leaders’ views of their practice in the multicultural care settings of the United Arab Emirates. Contemp. Nurse.

[67] Teixeira G., Gaspar F., Lucas P. (2024). Development and Validation of the Portuguese Transcultural Nursing Leadership Questionnaire (QLTE-PT). J. Nurs. Manag..

[68] Wong C.A. (2015). Connecting nursing leadership and patient outcomes: State of the science. J. Nurs. Manag..

[69] Wong C.A., Cummings G.G., Ducharme L. (2013). The relationship between nursing leadership and patient outcomes: A systematic review update. J. Nurs. Manag..

[70] Gunawan J., Aungsuroch Y., Fisher M.L. (2018). Factors contributing to managerial competence of first-line nurse managers: A systematic review. Int. J. Nurs. Pract..

[71] Cummings G., Tate K., Lee S., Wong C., Paananen T., Micaroni S., Chatterjee G. (2018). Leadership styles and outcome patterns for the nursing workforce and work environment: A systematic review. Int. J. Nurs. Stud..

[72] Honkavuo L., Lindström U.Å. (2014). Nurse leaders’ responsibilities in supporting nurses experiencing difficult situations in clinical nursing. J. Nurs. Manag..

[73] Fernet C., Gillet N., Austin S., Trépanier S.-G., Drouin-Rousseau S. (2021). Predicting nurses’ occupational commitment and turnover intention: The role of autonomous motivation and supervisor and coworker behaviours. J. Nurs. Manag..

[74] Matveev A. (2017). Intercultural Competence in Organizations: A Guide for Leaders, Educators and Team Players.

[75] AONE, AONL (2015). AONL Nurse Manager Competencies.

[76] Registered Nurses’ Association of Ontario (RNAO), RNAO (2013). Developing and Sustaining Nursing Leadership Best Practice Guideline.

[77] Pearson A., Laschinger H., Porritt K., Jordan Z., Tucker D., Long L. (2007). Comprehensive systematic review of evidence on developing and sustaining nursing leadership that fosters a healthy work environment in healthcare. Int. J. Evid. Based. Healthc..

[78] Campinha-Bacote J. (2002). The Process of Cultural Competence in the Delivery of Healthcare Services: A Model of Care. J. Transcult. Nurs..

[79] Dauvrin M., Lorant V. (2015). Leadership and cultural competence of healthcare professionals. Nurs. Res..

[80] Diversity & Cultural Competency in Health Care Settings. https://www.ecald.com/assets/Resources/Assets/Diversity-and-Cultural-Competency.pdf.

[81] Alizadeh S., Chavan M. (2016). Cultural competence dimensions and outcomes: A systematic review of the literature. Health Soc. Care Community.

[82] Almutairi A.F., Gardner G., McCarthy A. (2013). Perceptions of clinical safety climate of the multicultural nursing workforce in Saudi Arabia: A cross-sectional survey. Collegian.

[83] Suarez-Balcazar Y., Balcazar F., Taylor-Ritzler T., Portillo N., Rodakowsk J., Garcia-Ramirez M., Willis C. (2011). Development and Validation of the Cultural Competence Assessment Instrument: A Factorial Analysis. J. Rehabil..

[84] Lauring J., Selmer J. (2012). Openness to diversity, trust and conflict in multicultural organizations. J. Manag. Organ..

[85] Mosed H., Periord M., Caboral-Stevens M. (2021). A concept analysis of intercultural communication. Nurs. Forum.

[86] Alshammari M., Duff J., Guilhermino M. (2019). Barriers to nurse-patient communication in Saudi Arabia: An integrative review. BMC Nurs..

[87] Sherwood G.D., Shaffer F.A. (2014). The role of internationally educated nurses in a quality, safe workforce. Nurs. Outlook.

[88] Anders R.L. (2021). Practical tips for effective communication. Nurs. Manag..

[89] Ashikali T., Groeneveld S., Ritz A., Leisink P., Andersen L., Brewer G.A., Jacobsen C., Knies E., Vandenabeele W. (2021). Managing a diverse workforce. Managing for Public Service Performance: How HRM and Leadership Can Make a Difference.

[90] Mccutcheon A., Huber D. (2010). Confronting the nursing shortage. Leadership and Nursing Care Management.

[91] De Los Santos J.A.A., Rosales R.A., Falguera C.C., Firmo C.N., Tsaras K., Labrague L.J. (2020). Impact of organizational silence and favoritism on nurse’s work outcomes and psychological well-being. Nurs. Forum.

[92] Hanges P.J., Aiken J.R., Park J., Su J. (2016). Cross-cultural leadership: Leading around the world. Curr. Opin. Psychol..

