# Supplementary material for: Impact of Nurse Manager’s Attributes on Multi-Cultural Nursing Teams: A Scoping Review
Source: Nurs Rep. 2024 Jul 15;14(3):1676–92. doi: 10.3390/nursrep14030125 (PMC11487393; doi:10.3390/nursrep14030125)
Supplement: Supplementary file 1 [file nursrep-14-00125-s001.zip › Supplementary File S1.pdf]

Supplementary File S1. Full Search Strategy.

Database	Strategy	Search terms
CINAHL complete (by EBSCO)<br>Search date:<br><br>No geographical or publication dates limitations. Filters: abstract available, Portuguese, English and Spanish.	S1	(AB (nurs* leader* OR (MH "Nurs* Leader*") OR (MH "Leader*") OR nurs* manag* OR (MH "Nurs* Manage*") OR nurs* admin* OR (MH "Nurs* Administrat*")) OR "transcultural nurs* leader*")) OR (TI (nurs* leader* OR (MH "Nurs* Leader*") OR (MH "Leader*") OR nurs* manag* OR (MH "Nurs* Manage*") OR nurs* admin* OR (MH "Nurs* Administrat*") OR "transcultural nurs* leader*"))
	S2	(AB (competenc* OR skill* OR trait* OR behavio#r* OR style* OR (MH "Management Styles"))) OR (TI (competenc* OR skill* OR trait* OR behavio#r* OR style* OR (MH "Management Styles")))
	S3	(AB (nursing workgroup diversity OR multicultural staff OR multicultural nurs* OR multicultural nurs* work* OR (MH "Cultural Diversity") OR (MH "Leininger's Theory of Culture Care Diversity and Universality") OR cross-cultural OR multicultural environment OR multicultural care work* OR multiculturalism OR multi-cultural nurs* OR culturally diverse work* OR migrant nurs* OR expatriate nurs* OR oversea* nurs* OR (MH "foreign nurses"))) OR (TI (nursing workgroup diversity OR multicultural staff OR multicultural nurs* OR multicultural nurs* work* OR (MH "Cultural Diversity") OR (MH "Leininger's Theory of Culture Care Diversity and Universality") OR cross-cultural OR multicultural environment OR multicultural care work* OR multiculturalism OR multi-cultural nurs* OR culturally diverse work* OR migrant nurs* OR expatriate nurs* OR oversea* nurs* OR (MH "foreign nurses")))
	S4	(AB (nurs* outcome* OR (MH "Nursing Outcomes") OR racism OR violence OR discrimination OR (MM "Discrimination, Employment") OR (MH "Discrimination") OR (MH "Prejudice") OR (MH "Ageism") OR (MH "Racism") OR (MH "Sexism") OR (MH "Sexual Harassment") OR job satisfaction OR job dissatisfaction OR job performance OR (MH "Motivation") OR (MH "Job Performance") OR (MH "Job Satisfaction") OR (MH "Presenteeism") OR (MH "Productivity") OR (MH "Work-Life Balance") OR intention to leave OR job commitment OR (MH "Commitment") OR job engagement OR cultural conflict* OR cultural clash* OR cultural pain OR (MH "Acculturation") OR depression OR anxiety OR stress OR burnout OR (MH "Burnout, Professional+") OR turnover OR (MH "Personnel Turnover") OR teamwork OR (MH "Teamwork") OR quality of life OR (MH "Quality of Working Life") OR well-being OR (MH "Psychological Well-Being") OR culturally competent care OR culturally congruent care OR (MH "Cultural Competence") OR (MH "Transcultural Care") OR quality of care OR (MH "Quality of Nursing Care") OR (MH "Nurse Attitudes") OR (MH "Teamwork"))) OR (TI (nurs* outcome* OR (MH "Nursing Outcomes") OR racism OR violence OR discrimination OR (MM "Discrimination, Employment") OR (MH "Discrimination") OR (MH "Prejudice") OR (MH "Ageism") OR (MH "Racism") OR (MH "Sexism") OR (MH "Sexual Harassment") OR job satisfaction OR job dissatisfaction OR job performance OR (MH "Motivation") OR (MH "Job Performance") OR (MH "Job Satisfaction") OR (MH "Presenteeism") OR (MH "Productivity") OR (MH "Work-Life Balance") OR intention to leave OR job commitment OR (MH "Commitment") OR job engagement OR cultural conflict* OR cultural clash* OR cultural pain OR (MH "Acculturation") OR depression OR anxiety OR stress OR burnout OR (MH "Burnout, Professional+") OR turnover OR (MH "Personnel Turnover") OR teamwork OR (MH "Teamwork") OR quality of life OR (MH "Quality of Working Life") OR well-being OR (MH "Psychological Well-Being") OR culturally competent care OR culturally congruent care OR (MH "Cultural Competence") OR (MH "Transcultural Care") OR quality of care OR (MH "Quality of Nursing Care") OR (MH "Nurse Attitudes") OR (MH "Teamwork")))
	S5	S1 AND S2 AND S3 AND S4
MEDLINE complete (by EBSCO)<br>Search date:<br><br>No geographical or publication dates limitations. Filters: abstract available, Portuguese, English and Spanish.	S1	(AB (nurs* leader* OR (MH "Leader*") OR nurs* manage* OR nurs* administrat* OR (MH "Nurs* Administrat*") OR transcultural leader*)) OR (TI (nurs* leader* OR (MH "Leader*") OR nurs* manage* OR nurs* administrat* OR (MH "Nurs* Administrat*") OR transcultural leader*))
	S2	(AB (skill* OR competenc* OR trait* OR behavio#r* OR style* OR (MH "Personnel Management"))) OR (TI (skill* OR competenc* OR trait* OR behavio#r* OR style* OR (MH "Personnel Management")))
	S3	(AB (multicultural nursing workforce OR multicultural nurs* OR cultural diversity OR internationality OR (MH "Cultural Diversity") OR migrant nurse* OR (MH "Nurses, International") OR expatriate nurs* OR overs* nurs* OR (MH "foreign nurses"))) OR (TI (multicultural nursing workforce OR multicultural nurs* OR cultural diversity OR internationality OR (MH "Cultural Diversity") OR migrant nurse* OR (MH "Nurses, International") OR expatriate nurs* OR overs* nurs* OR (MH "foreign nurses")))
	S4	(AB (nurs* outcome* OR violence OR (MH "Workplace Violence") OR (MH "Ageism") OR (MH "Racism") OR (MH "Prejudice") OR (MH "Sexism") OR (MH "Social Discrimination") OR discrimination OR (MH "Sexual Harassment") OR job satisfaction OR job dissatisfaction OR (MH "Job Satisfaction") OR

		job performance OR (MH "Work Performance") OR job engagement OR (MH "Work Engagement") OR job commitment OR motivation OR (MH "Motivation") OR (MH "Presenteeism") OR productivity OR (MH "Absenteeism") OR (MH "Efficiency") OR (MH "Work-Life Balance") OR intention to leave OR (MH "Personnel Turnover") OR cultural conflict* OR cultural clash* OR cultural pain OR (MH "Acculturation") OR depression OR anxiety OR stress OR burnout OR (MH "Depression") OR (MH "Stress, Psychological") OR (MH "Burnout, Professional") OR teamwork OR (MH "Quality of Life") OR well-being OR (MH "Culturally Competent Care") OR (MH "Cultural Competency") OR culturally congruent care OR transcultural care OR quality of care OR quality of nurs* care)) OR (TI (nurs* outcome* OR violence OR (MH "Workplace Violence") OR (MH "Ageism") OR (MH "Racism") OR (MH "Prejudice") OR (MH "Sexism") OR (MH "Social Discrimination") OR discrimination OR (MH "Sexual Harassment") OR job satisfaction OR job dissatisfaction OR (MH "Job Satisfaction") OR job performance OR (MH "Work Performance") OR job engagement OR (MH "Work Engagement") OR job commitment OR motivation OR (MH "Motivation") OR (MH "Presenteeism") OR productivity OR (MH "Absenteeism") OR (MH "Efficiency") OR (MH "Work-Life Balance") OR intention to leave OR (MH "Personnel Turnover") OR cultural conflict* OR cultural clash* OR cultural pain OR (MH "Acculturation") OR depression OR anxiety OR stress OR burnout OR (MH "Depression") OR (MH "Stress, Psychological") OR (MH "Burnout, Professional") OR teamwork OR (MH "Quality of Life") OR well-being OR (MH "Culturally Competent Care") OR (MH "Cultural Competency") OR culturally congruent care OR transcultural care OR quality of care OR quality of nurs* care))
	S5	S1 AND S2 AND S3 AND S4
Nursing & Allied Health Collection<br>Search date:<br><br>No geographical or publication dates limitations. Filters: abstract available, Portuguese, English and Spanish.	S1	[All] (DE "NURS* administrat*" OR nurs* leader* OR nurs* manage* OR nurs* administrat*)
	S2	[All] (DE "AUTHENTIC leadership" OR DE "DEVELOPMENT leadership" OR DE "INCLUSIVE leadership" OR DE "LEADER-member exchange theory" OR DE "SERVANT leadership" OR DE "SITUATIONAL leadership theory" OR DE "TRANSACTIONAL leadership" OR DE "TRANSFORMATIONAL leadership" OR DE "CONTINGENCY theory (Management)" OR DE "MANAGEMENT styles" OR skill* OR competenc* OR trait* OR behavio#r* OR style*)
	S3	[All] ((DE "DIVERSITY in the workplace") OR (DE "FOREIGN nurse*") OR foreign nurs* OR expatriate nurs* OR oversea* nurs* OR international nurs* OR migrant nurs*)
	S4	[All] ((((((((((((((((((((DE "PREJUDICES" OR DE "AGE discrimination")) OR (DE "RACISM in the workplace")) AND (DE "PREJUDICES" OR DE "CULTURAL prejudices")) AND (DE "SEX discrimination" OR DE "GENDER inequality" OR DE "SEXUAL harassment")) AND (DE "DISCRIMINATORY language" OR DE "EMPLOYMENT discrimination" OR DE "ETHNIC discrimination" OR DE "INDIRECT discrimination" OR DE "RACE discrimination" OR DE "RELIGIOUS discrimination" OR DE "SEGREGATION" OR DE "MINORITIES" OR DE "PERSECUTION" OR DE "STEREOTYPES" OR DE "XENOPHOBIA")) OR (DE "JOB satisfaction")) AND (DE "PERFORMANCE" OR DE "TASK performance")) OR (DE "ORGANIZATIONAL commitment")) OR (DE "EMPLOYEE motivation")) OR (DE "PRESENTEEISM (Labor)")) OR (DE "LABOR productivity")) OR (DE "JOB absenteeism")) OR (DE "WORK-life balance")) OR (DE "LABOR turnover")) AND (DE "CULTURE conflict" OR DE "CULTURE shock")) OR (DE "ACCULTURATION")) OR (DE "ANXIETY")) OR (DE "JOB stress")) OR (DE "TEAM nursing")) OR (DE "WELL-being")) AND (DE "CULTURALLY competent medical care" OR DE "CULTURAL competence" OR DE "TRANSCULTURAL medical care")) OR (DE "QUALITY of work life") OR nurs* outcome* OR workplace violence OR intention to leave OR cultural clash* OR cultural pain OR depression OR burnout OR teamwork OR quality of care OR quality of nurs* care OR (DE "VIOLENCE"))
	S5	S1 AND S2 AND S3 AND S4
Cochrane Database of Systematic Reviews<br>Search date:<br><br>No geographical or publication dates limitations. Filters: abstract available, Portuguese, English and Spanish.	#1	[All] (nurs* leader* OR (MH "Leadership") OR nurs* manage* OR nurs* administrat* OR (MH "Nurs* Administrat*"))
	#2	[All] (skill* OR competenc* OR trait* OR behavio#r* OR style*)
	#3	[All] (multicultural nursing workforce OR multicultural nurs* OR cultural diversity OR internationality OR (ZU "cultural diversity") OR migrant nurs* OR expatriate nurs* OR oversea* nurs*)
	#4	[All] (nurs* outcome* OR racism OR discrimination OR ageism OR sexism OR sexual harassment OR dissatisfaction OR performance OR presenteeism OR productivity OR work-life balance OR intention to leave OR commitment OR engagement OR conflict OR cultural clashes OR cultural pain OR acculturation OR stress OR turnover OR teamwork OR well-being OR transcultural care OR quality of nurs* care OR nurse attitudes OR teamwork OR ((((((((((((ZU "violence")) OR ((ZU "prejudice"))) OR ((ZU "job satisfaction"))) OR ((ZU "motivation"))) OR ((ZU "professional autonomy") OR (ZU "professional

			competence"))) OR ((ZU "depression"))) OR ((ZU "anxiety"))) OR ((ZU "burnout, professional"))) OR ((ZU "quality of life"))) OR ((ZU "culturally competent care"))) OR ((ZU "cultural competency"))) OR ((ZU "quality of health care")))
		#5	S1 AND S2 AND S3 AND S4
MedicLatina (by EBSCO)<br>Search date:<br><br>No geographical or publication dates limitations. Filters: abstract available, Portuguese, English and Spanish.		S1	TX (nurs* leader* OR nurs* manage* OR nurs* administrat* OR transcultural leader*)
		S2	TX (competenc* OR skill* OR trait* OR behavio#r* OR style*)
		S3	TX (nurs* workgroup diversity OR multicultural staff OR multicultural nurs* OR cultural diversity OR migrant nurs* OR expatriate nurs* OR oversea* nurs* OR foreign nurs* OR multicultural nurs* workforce OR international nurs*)
		S4	TX (nurs* outcome* OR racism OR violence OR discrimination OR prejudice OR ageism OR sexism OR sexual harassment OR job satisfaction OR dissatisfaction OR performance OR efficiency OR motivation OR absenteeism OR presenteeism OR productivity OR work-life balance OR intention to leave OR commitment OR engagement OR cultural conflict* OR cultural clashe* OR cultural pain OR acculturation OR depression OR anxiety OR stress OR burnout OR turnover OR teamwork OR quality of life OR well-being OR culturally competent care OR culturally congruent care OR cultural competence OR transcultural care OR quality of care OR quality of nursing care OR nurs* attitude* OR motivation)
		S5	S1 AND S2 AND S3 AND S4
Psychology and Behavioral Sciences Collection (by EBSCO)<br>Search date:<br><br>No geographical or publication dates limitations. Filters: abstract available, Portuguese, English and Spanish.		S1	TX (nurs* leader* OR nurs* manage* OR nurs* administrat* OR transcultural leader* OR (DE "NURS* administrat*") OR (DE "LEADER*")))
		S2	TX ((competenc* OR skill* OR trait* OR behavio#r* OR style*) AND (DE "LEADERSHIP")) OR (DE "MANAGEMENT styles")
		S3	TX (nurs* AND ((DE "DIVERSITY in organizations") OR (DE "DIVERSITY in the workplace")) OR overseas nurs* OR migrant nurse* OR expatriate nurs* OR international nurs* OR (DE "FOREIGN nurses")
		S4	TX ((((((((((((((((((((((DE "RACISM") OR (DE "VIOLENCE")) OR (DE "DISCRIMINATION")) OR (DE "PREJUDICES")) OR (DE "PREJUDICES -- Religious aspects")) OR (DE "CULTURAL prejudices")) OR (DE "AGEISM")) AND (DE "PREJUDICES" OR DE "AGE discrimination" OR DE "AGE stereotypes")) OR (DE "PREJUDICES" OR DE "SEX discrimination" OR DE "GENDER inequality")) OR (DE "SEXUAL harassment of men" OR DE "SEXUAL harassment of women" OR DE "GENDER role in the work environment")) OR (DE "JOB satisfaction of nurses")) OR (DE "TASK performance")) OR (DE "JOB absenteeism")) AND (DE "PRESENTEEISM (Labor)" OR DE "WORKING hours" OR DE "ATTENDANCE" OR DE "JOB absenteeism" OR DE "LEAVE of absence" OR DE "PRESENTEEISM (Labor)" OR DE "SICK leave")) OR (DE "WORK-life balance")) OR (DE "CULTURE conflict" OR DE "CULTURAL exclusion" OR DE "SOCIAL marginality")) OR (DE "ACCULTURATION" OR DE "CULTURAL imperialism" OR DE "CULTURE conflict")) OR (DE "ANXIETY")) OR (DE "NURSING" OR DE "ADDICTIONS nursing" OR DE "COMMUNICATION in nursing" OR DE "MINORITIES in nursing" OR DE "TEAM nursing" OR DE "TRANSCULTURAL nursing" OR DE "TRAVEL nursing" OR)) OR (DE "QUALITY of life")) OR (DE "WELL-being")) OR (DE "CULTURAL competence")) OR (DE "TRANSCULTURAL medical care" OR DE "CULTURALLY competent medical care" OR DE "TRANSCULTURAL nursing") OR nurs* outcome* OR dissatisfaction OR motivation OR intention to leave OR commitment OR depression OR stress OR burnout OR turnover OR culturally competent care OR culturally congruent care OR quality of care OR quality of nurs* care OR nurs* attitude*)
		S5	S1 AND S2 AND S3 AND S4
Wiley Online Library<br>Search date:<br><br>No geographical or publication dates limitations. Filters: abstract available, Portuguese, English and Spanish.		S1	[All] (nurs* leader* OR nurs* manage* OR nurs* administrat* OR transcultural leader*)
		S2	[All] (competenc* OR skill* OR trait* OR behavio*r* OR style*)
		S3	[All] (nurs* workgroup diversity OR multicultural staff OR multicultural nurs* OR cultural diversity OR migrant nurs* OR expatriate nurs* OR oversea* nurs* OR foreign nurs* OR multicultural nurs* workforce OR international nurs*)
		S4	[All] (nurs* outcome* OR racism OR violence OR discrimination OR prejudice OR ageism OR sexism OR sexual harassment OR job satisfaction OR dissatisfaction OR performance OR efficiency OR motivation OR absenteeism OR presenteeism OR productivity OR work-life balance OR intention to leave OR commitment OR engagement OR cultural conflict* OR cultural clashe* OR cultural pain OR

	S5	acculturation OR depression OR anxiety OR stress OR burnout OR turnover OR teamwork OR quality of life OR well-being OR culturally competent care OR culturally congruent care OR cultural competence OR transcultural care OR quality of care OR quality of nurs* care OR nurs* attitude*)<br><br>S1 AND S2 AND S3 AND S4
SciELO<br>Search date:<br><br>No geographical or publication dates limitations. Filters: abstract available, Portuguese, English and Spanish.	S1<br><br>S2<br><br>S3<br><br><br><br>S4<br><br><br><br><br><br><br><br>S5	[All] (nurs* leader* OR nurs* manage* OR nurs* administrat*)<br><br>[All] (competenc* OR skill* OR trait* OR behavior* OR style*)<br><br>[All] (nurs* workgroup diversity OR multicultural staff OR multicultural nurs* OR cultural diversity OR migrant nurs* OR expatriate nurs* OR oversea* nurs* OR foreign nurs* OR multicultural nurs* workforce OR international nurs*)<br><br>[All] (nurs* outcome* OR racism OR violence OR discrimination OR prejudice OR ageism OR sexism OR sexual harassment OR job satisfaction OR dissatisfaction OR performance OR efficiency OR motivation OR absenteeism OR presenteeism OR productivity OR work-life balance OR intention to leave OR commitment OR engagement OR cultural conflict* OR cultural clashe* OR cultural pain OR acculturation OR depression OR anxiety OR stress OR burnout OR turnover OR teamwork OR quality of life OR well-being OR culturally competent care OR culturally congruent care OR cultural competence OR transcultural care OR quality of care OR quality of nursing care OR nurs* attitude* OR motivation)<br><br>S1 AND S2 AND S3 AND S4
LILACS<br>Search date:<br><br>No geographical or publication dates limitations. Filters: abstract available, Portuguese, English and Spanish.	S1<br><br>S2<br><br><br>S3<br><br><br><br><br><br><br><br>S4	(nurse leader OR nurse manager OR nurse administrator)<br><br>(cultural diversity OR multicultural OR foreign nurses OR international nurses OR migrant nurses OR expatriate nurses OR overseas nurses)<br><br>(racism OR violence OR discrimination OR prejudice OR ageism OR sexism OR sexual harassment OR job satisfaction OR dissatisfaction OR performance OR efficiency OR motivation OR absenteeism OR presenteeism OR productivity OR work-life balance OR intention to leave OR commitment OR engagement OR cultural conflicts OR cultural clashes OR cultural pain OR acculturation OR depression OR anxiety OR stress OR burnout OR turnover OR teamwork OR quality of life OR well-being OR culturally competent care OR culturally congruent care OR cultural competence OR transcultural care OR quality of care OR quality of nursing care OR nurses attitudes OR motivation)<br><br>S1 AND S2 AND S3
Scopus<br>Search date:<br><br>No geographic or publication dates limitations. Filters: abstract available, Portuguese, English and Spanish.		(ALL (nurse leader OR nurse manager OR nurse administrator) AND (cultural diversity OR multicultural OR foreign nurses OR international nurses OR migrant nurses OR expatriate nurses OR overseas nurses) AND (racism OR violence OR discrimination OR prejudice OR ageism OR sexism OR sexual harassment OR job satisfaction OR dissatisfaction OR performance OR efficiency OR motivation OR absenteeism OR presenteeism OR productivity OR work-life balance OR intention to leave OR commitment OR engagement OR cultural conflicts OR cultural clashes OR cultural pain OR acculturation OR depression OR anxiety OR stress OR burnout OR turnover OR teamwork OR quality of life OR well-being OR culturally competent care OR culturally congruent care OR cultural competence OR transcultural care OR quality of care OR quality of nursing care OR nurses attitudes OR motivation))
OpenGrey<br>Search date:<br><br>No geographical or publication dates limitations. Filters: abstract available, Portuguese, English and Spanish.		(nurs* leader* OR nurs* manage* OR nurs* administrat* OR transcultural leader*) AND (competenc* OR skill* OR trait* OR behavio#r* OR style*) AND (nurs* workgroup diversity OR multicultural staff OR multicultural nurs* OR cultural diversity OR migrant nurs* OR expatriate nurs* OR oversea* nurs* OR foreign nurs* OR multicultural nurs* workforce OR international nurs*) AND (nurs* outcome* OR racism OR violence OR discrimination OR prejudice OR ageism OR sexism OR sexual harassment OR job satisfaction OR dissatisfaction OR performance OR efficiency OR motivation OR absenteeism OR presenteeism OR productivity OR work-life balance OR intention to leave OR commitment OR engagement OR cultural conflict* OR cultural clashe* OR cultural pain OR acculturation OR depression OR anxiety OR stress OR burnout OR turnover OR teamwork OR quality of life OR well-being OR culturally competent care OR culturally congruent care OR cultural competence OR transcultural care OR quality of care OR quality of nurs* care OR nurs* attitude* OR motivation)
Google Scholar<br><br>Search date:<br><br>No geographical or publication dates limitations. Filters:		(nurs* leader* OR nurs* manage* OR nurs* administrat* OR transcultural leader*) AND (competenc* OR skill* OR trait* OR behavio#r* OR style*) AND (nurs* workgroup diversity OR multicultural staff OR multicultural nurs* OR cultural diversity OR migrant nurs* OR expatriate nurs* OR oversea* nurs* OR foreign nurs* OR multicultural nurs* workforce OR international nurs*) AND (nurs* outcome* OR racism OR violence OR discrimination OR prejudice OR ageism OR sexism OR sexual harassment OR job satisfaction OR dissatisfaction OR performance OR efficiency OR motivation OR absenteeism OR presenteeism OR productivity OR work-life balance OR intention to leave OR commitment OR engagement

abstract available, Portuguese, English and Spanish.		OR cultural conflict* OR cultural clashe* OR cultural pain OR acculturation OR depression OR anxiety OR stress OR burnout OR turnover OR teamwork OR quality of life OR well-being OR culturally competent care OR culturally congruent care OR cultural competence OR transcultural care OR quality of care OR quality of nurs* care OR nurs* attitude* OR motivation)
RCAAP<br>Search date:<br><br>No geographic or publication dates limitations. Filters: abstract available, Portuguese, English and Spanish.		(nurse leader OR nurse manager OR nurse administrator OR nursing leadership OR nursing management OR nursing administration) AND (cultural diversity OR multicultural team OR foreign nurses OR international nurses OR migrant nurses OR expatriate nurses OR overseas nurses) AND (racism OR violence OR discrimination OR prejudice OR ageism OR sexism OR sexual harassment OR job satisfaction OR dissatisfaction OR performance OR efficiency OR motivation OR absenteeism OR presenteeism OR productivity OR work-life balance OR intention to leave OR commitment OR engagement OR cultural conflicts OR cultural clashes OR cultural pain OR acculturation OR depression OR anxiety OR stress OR burnout OR turnover OR teamwork OR quality of life OR well-being OR culturally competent care OR culturally congruent care OR cultural competence OR transcultural care OR quality of care OR quality of nursing care OR nurses attitudes OR motivation)
