# Supplementary material for: Impact of Nurse Manager’s Attributes on Multi-Cultural Nursing Teams: A Scoping Review
Source: Nurs Rep. 2024 Jul 15;14(3):1676–92. doi: 10.3390/nursrep14030125 (PMC11487393; doi:10.3390/nursrep14030125)
Supplement: Supplementary file 1 [file nursrep-14-00125-s001.zip › Supplementary File S2.pdf]

Supplementary File S2. Nurse managers' personality traits impact on nurses' outcomes in multicultural teams.

Nurse managers' personality traits	Nurses' outcomes										
	Job satisfaction	OCB	Retention	SPI	Autonomy	Achievement of common goals	Interpersonal relationships	CCC	Discrimination	Coping stress factors	CSCWE
Supportive	+	+	+	+							
Cooperative	+							+			
Understanding	+										
Fair	+										
Proactive	+										
Unbiased	+			+		+	+				
Sensitive			+				+				
Approachable			+	+				+			
Respectful			+								
Nurses advocate			+								
Receptive to people and ideas			+								
Compassionate				+							
Empathetic				+							
Genuine				+							
Thoughtful				+							
Trustworthy				+				+			
Rigorous				+							
Insightful							+				
Focused					+	+					
Visionary					+	+					
Flexible								+			
Good listener								+			
Courageous								+			
Self-confident								+	+		
Responsible								+			
Appreciative										+	
Aware of personal biases											+
Knowledgeable of personal cultural preferences											+
Biased	-										

Note: '+' means positive impact; '-' means negative impact

Abbreviations: CCC - Culturally congruent care; CSCWE - Culturally safe & competent work environment; OCB - Organizational citizenship behaviours; SPI - Sociocultural and professional integration
