# Supplementary material for: Impact of Nurse Manager’s Attributes on Multi-Cultural Nursing Teams: A Scoping Review
Source: Nurs Rep. 2024 Jul 15;14(3):1676–92. doi: 10.3390/nursrep14030125 (PMC11487393; doi:10.3390/nursrep14030125)
Supplement: Supplementary file 1 [file nursrep-14-00125-s001.zip › Supplementary File S3.pdf]

Supplementary File S3. Impact of nurse managers' competences on nurses' outcomes in multicultural teams.

Nurse managers' competences	Nurses' outcomes														
	Job satisfaction	OCB	Organizational commitment	Burnout	Retention	SPI	Autonomy	Achievement common goals	Interpersonal relationships	CCC	Discrimination	Performance	Innovation & productivity	Teamwork	CSCWE
Cultural competence		+			+	+		+	+	+			+	+	+
Effective communication	+		+					+	+	+	+			+	
Leadership skills	+	+	+	+	+	+	+	+		+					
Diversity management						+									
Problem & conflict solving	+								+			+			+
Relational skills	+					+									
Coaching						+									
Supervision												+			
Clinical expertise						+									

Note: '+' means positive impact

Abbreviations: CCC - Culturally congruent care; CSCWE - Culturally safe & competent work environment; OCB - Organizational citizenship behaviours; SPI - Sociocultural and professional integration
