# Supplementary material for: Impact of Nurse Manager’s Attributes on Multi-Cultural Nursing Teams: A Scoping Review
Source: Nurs Rep. 2024 Jul 15;14(3):1676–92. doi: 10.3390/nursrep14030125 (PMC11487393; doi:10.3390/nursrep14030125)
Supplement: Supplementary file 1 [file nursrep-14-00125-s001.zip › Supplementary File S4.pdf]

Supplementary File S4. Impact of nurse managers' behaviours on nurses' outcomes in multicultural teams.

**Nurse managers' behaviours**	**Nurses' outcomes**																	
	Job satisfaction	Organizational commitment	Burnout	Retention	SPI	Professional development	Competence development	Teamwork	Common goals	Performance	Innovation & productivity	Interpersonal relationships	CCC	Quality of care	Discrimination	Coping stress	CSCWE	Well-being
Appreciating & recognizing	+				+					+		+	+			+		
Improving practice	+				+	+				+		+	+					
Sharing governance	+				+					+								
Advancing nurses' career	+					+						+	+				+	
Integrating in the work environment		+			+		+	+										+
Promoting psychosocial well-being at work	+				+			+				+	+		-		+	
Dynamizing groups	+				+							+	+					
Communicating	+				+				+			+				+		
Coordinating nursing care	+		+									+	+	+				
Downgrading	-			-				-			-				+			
Lacking management performance	-																	
Centralizing governance	-			-														
Favouring	-			-		-		-			-				+			
Hampering nurses' career advancement						-									+			

Note: '+' means the behaviours increase the outcome; '-' means the behaviours reduce the outcome
Abbreviations: CCC - Culturally congruent care; CSCWE - Culturally safe & competent work environment; SPI - Sociocultural and professional integration
